# Efficacy and Safety of Using Antifibrinolytic Agents in Spine Surgery: a Meta-Analysis

**DOI:** 10.1371/journal.pone.0082063

**Published:** 2013-11-22

**Authors:** Chaoqun Yuan, Hailong Zhang, Shisheng He

**Affiliations:** Department of Orthopaedics, Shanghai Tenth People’s Hospital, Tongji University School of Medicine, Shanghai, China; National Cerebral and Cardiovascular Center, Japan

## Abstract

**Purpose:**

Spine surgery, particularly reconstructive surgery, can be associated with significant blood loss, and blood transfusion. Antifibrinolytic agents are used routinely to reduce bleeding in cardiac, orthopaedic, and hepatic surgery. The purpose of this study was to assess the efficacy and safety of using antifibrinolytic agents in reducing blood loss and blood transfusions in spine surgery.

**Methods:**

A systematic search of all related studies written in English published by October 2012 was conducted using the MEDLINE, EMBASE and the Cochrane Library databases. Randomized controlled trials that reported the drug dosage, total blood loss, blood transfusion and incidence of deep vein thrombosis as the primary outcome were included.

**Results:**

Nine studies involving 482 patients were identified. Patients receiving antifibrinolytic agents had reduced blood loss (WMD =-288.8, 95 % CI – 46.49, - 110.19; *P* = 0.002), reduced blood transfusion (WMD =-242.7, 95 % CI – 422.57, - 62.95; *P* = 0.008), reduced blood transfusion rate (RR 0.73, 95% CI 0.58, 0.93; *p* = 0.010) and no increase (RR 0.25, 95 % CI 0.03, 2.22; *P* = 0.21) in the risk of deep vein thrombosis.

**Conclusions:** We conclude that antifibrinolytic agents significantly decrease blood loss, blood transfusion, and there is no increase in the risk of deep vein thrombosisfor transfusion requirements in spine surgery.

## Introduction

Spine surgery has typically been associated with significant blood loss and transfusion requirements. It is particularly common for multilevel spinal fusion [1], deformity correction [2] and anterior-posterior spinal fusion [3]. Although blood transfusions may effectively replace perioperative blood loss, there is a potential for transfusion reactions/complications and disease transmission [4]. Data further suggests that both bleeding and resultant transfusions are associated with an increased risk of adverse outcomes [5]. Measures to decrease transfusion-related complications such as preoperative autologous blood donation, application of cell saver-systems or the use of erythropoietin are often associated with higher costs and logistic challenges [6-8].

Since the 1990s, intraoperative administration of antifibrinolytics has gained popularity as a means to control blood loss [9].There are various reports on the use of antifibrinolytic drugs, like tranexamic acid (TXA), epsilon-aminocaproic acid (EACA), and aprotinin to reduce the blood loss and transfusion requirements in spine surgery. In 2008, Gill JB et al. [10] had performed a meta-analysis of prospective clinical trials to assess whether antifibrinolytic agents (TXA, EACA, Aprotinin) reduce bleeding and transfusion requirements in patients undergoing spine surgery. But the main limitation of that meta-analysis is the quality of the studies included. As more high quality Randomized controlled trials were published, we therefore performed this meta-analysis of RCTs to check if antifibrinolytic agents reduced blood loss and blood transfusions in patients undergoing spine surgery, as well as their effect on the incidence of DVT.

## Materials and Methods

### Search strategy

Computerised search of the electronic databases MEDLINE, EMBASE and the Cochrane Library databases were performed for all studies written in English published by October 2012 that compared antifibrinolytic agents with placebo for sipne surgery. The following search terms were used to maximize the search specificity and sensitivity: spine surgery, spinal surgery, antifibrinolytic agents, tranexamic acid, and epsilon-aminocaproic acid. Secondary searches of the unpublished literature were conducted by searching the WHO International Clinical Trials Registry Platform, UK National Research Register Archive and Current Controlled Trials from their inception to October 2012. The reference lists of all the full-text papers were examined to identify any initially omitted studies.

### Inclusion Criteria

Studies were included if they met the following criteria: randomized controlled trials on spine surgery in which tranexamic acid or epsilon-aminocaproic acid was compared with placebo; outcomes: reported at least one of blood loss, blood transfusion, ratio of blood transfusion, incidence of DVT(deep vein thrombosis). Two reviewers independently screened the titles and abstracts for the eligibility criteria. Consensus was reached by discussion.

### Data extraction

Two of the authors independently extracted the following data from each full-text report using a standard data extraction form. The data extracted from studies included authors, year of publication, country, sample size, age, gender, drug dosage, transfusion indication, duration of surgery, total blood loss, blood transfusion, ratio of blood tansfusion, and incidence of DVT.

### Assessment of methodological quality

Following the Cochrane Handbook for Systematic Reviews of Interventions 5.0, the methodological quality of the included studies was independently assessed by two authors. Any disagreements were resolved by discussion. The corresponding author was the adjudicator when no consensus could be achieved. We evaluated the risk of bias of included studies using the Review Manager software (RevMan Version 5.2; The Nordic Cochrane Center, The Cochrane Collaboration, Copenhagen, Denmark),which included the following key domains: Random sequence generation (selection bias); Allocation concealment (selection bias); Blinding of participants and personnel (performance bias); Blinding of outcome assessment (detection bias); Incomplete outcome data (attrition bias); Selective reporting (reporting bias). The publication bias was assessed with funnel plots.

### Data analysis

We performed all of the meta-analyses with the Review Manager software (RevMan Version 5.2; The Nordic Cochrane Center, The Cochrane Collaboration, Copenhagen, Denmark). For continuous outcomes, such as total blood loss and blood tansfusion were pooled to a weighted mean difference (WMD) and 95 % confidence interval (CI). Risk ratios (RRs) and 95 % confidence intervals (CIs) were used to evaluate the dichotomous outcomes, such as ratio of blood tansfusion and incidence of DVT. A P value < 0.05 was considered to be statistically significant.

 The fixed effect model was used when the test for homogeneity was significant (*p*> 0. 05), while a P value of <0.05 was considered suggestive of statistical heterogeneity and random effect model was used. The sensitivity analysis was performed by rejecting the studies with higher statistical heterogeneity.

## Results

### Search results

**Figure 1:**
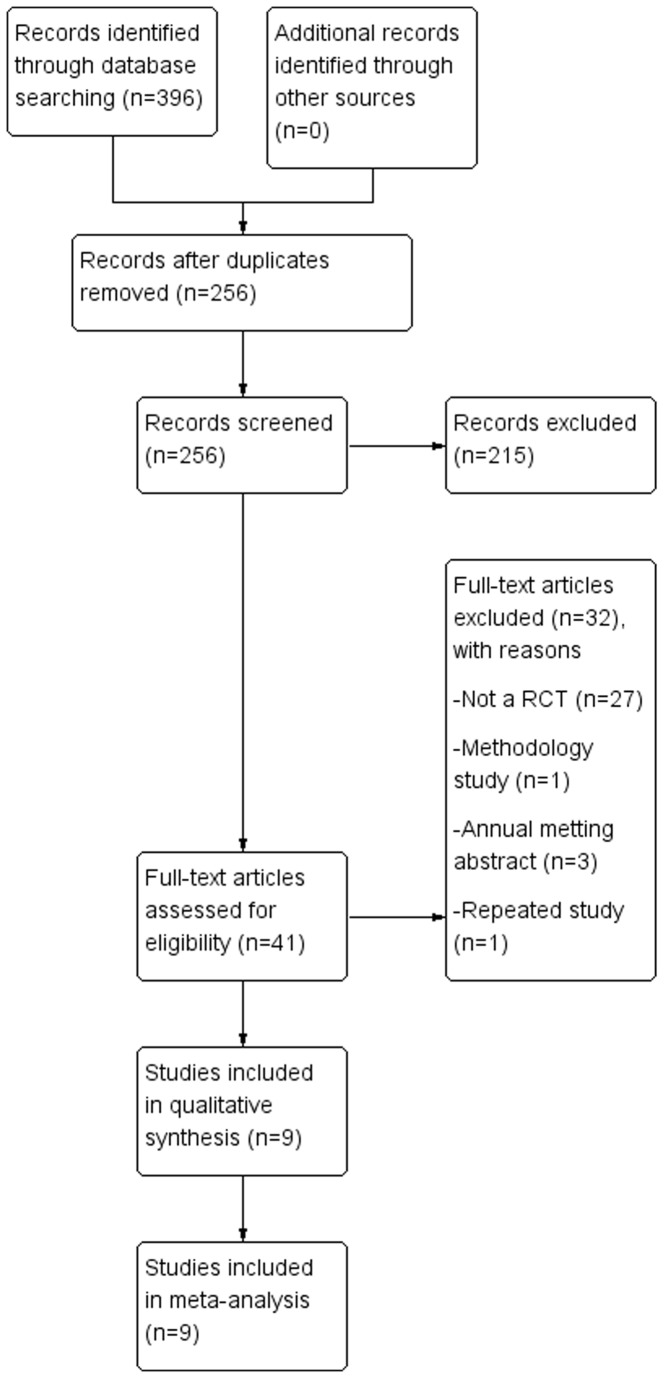
The study selection and inclusion process.

A total of 296 titles and abstracts were preliminarily reviewed, of which nine studies [3,11–18] eventually satisfied the eligibility criteria. The study selection process was summarised in Figure 1. These studies were all randomized controlled studies. 

### Study Characteristics and Quality

**Figure 2:**
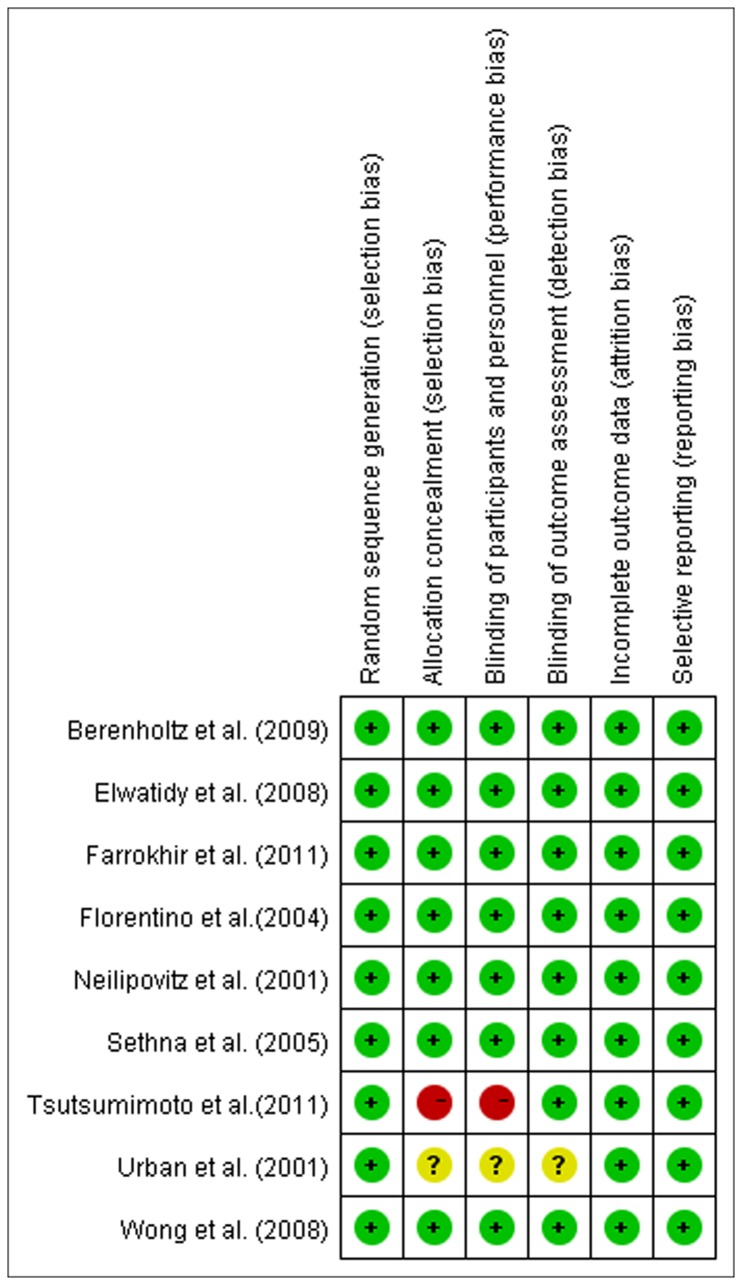
The risk of bias of the included studies.

Nine Randomized controlled trials directly comparing antifibrinolytic agents with placebo were included in this meta-analysis. All of the included studies had defined eligibility criteria. The baseline information of the studies without significant difference between these two groups is summarised in Table 1. These studies were evaluated with the risk of bias and the outcome was shown in Figure 2.

**Table 1:** Description of the studies included in the meta-analysis.

**First author, year**	**Country**	**F/M**	**Age (yr): experiment/control**	**Drug dosage**	**Transfusion indications**	**Useof anticoagulants**
Sethna [11], 2005	America	14/30	13.6/14.0	TXA: 100 mg/kg + 10 mg/kg/h(until skin closure)	HCT<25%	N/A
Wong [12], 2008	Canada	100/47	56.82/50.0	TXA: 10 mg/kg + 1 mg/kg/h(until skin closure)	Hb<70g/L	excluded
Elwatidy [13], 2008	Saudi Arabia	25/39	51.56/49.75	TXA: 2 g±100 mg/h(adults) or 30 mg/kg±1 mg/kg/h (for children)(until 5 hours after the operation	Hb<90g/L or HCT<27%	N/A
Tsutsumimoto [14], 2011	Japan	9/31	68.0/65.8	TXA:15mg/kg intravenously over 15 min before the surgery	N/A	excluded
Neilipovitz [3], 2001	Canada	23/17	14.1/13.7	TXA: 10 mg/kg + 1 mg/kg/h(until skin closure)	Hb<70g/L	N/A
Farrokhir [15], 2011	Iran	58/18	45.5/51.4	TXA: 10 mg/kg + 1 mg/kg/h(until skin closure)	Hb<100g/L	excluded
Florentino-Pineda [16], 2004	America	27/9	13.5/14.5	EACA:100 mg/kg + 10 mg/kg/h(until skin closure)	Hb<70g/L	N/A
Urban [17], 2001	America	35	46.6/47.3	EACA: 5 g/kg + 15 mg/kg/h (half full-dose regimen)	Hb<80g/L or HCT<25%	N/A
Berenholtz [18], 2009	America	127/55	55.5/55.4	EACA: 100 mg/kg 10 mg / kg / hr for 8 hours postoperative	Hb<80g/L	excluded

Note: SS, sample size; TXA, tranexamic acid; EACA, Amicar, epsilon-aminocaproic acid; HCT, hematocrit; Hb, hemoglobin; F/M, female/male; N/A, not available.

In total, 664patients were included in the 9 studies, and 335 patients received antifibrinolytic agents. The individual sample sizes ranged from 36 to 182 patients. Patients in all these studies received major spinal surgery. Except one study [3], patients in all groups received a dose of antifibrinolytic agents before anesthesia, and a maintenance dose continued until skin closure [3,11,12,15-17]or several hours after surgery[13,18]. However, the studies did differ in their doses of antifibrinolytic agents. The authors of five studies specified the protocol for estimating blood loss, which involved weighing the sponges in addition to estimating blood loss through suction drainage systems and estimating the amount of blood on the surgical drapes and gowns [3,11,15-17].In three other studies, blood loss was estimated in the same way, with the exception of that the investigators did not estimate the amount of blood on the surgical drapes and gowns [12-14]. In six studies, the investigators estimated the postoperative blood loss was measured from wound drainage of the surgical drain [3,12-14,16,17]. In Berenholtz’s [18] study, the method for calculating estimated blood loss was not specified. 

### Outcome analysis

#### Total blood loss

**Figure 3:**
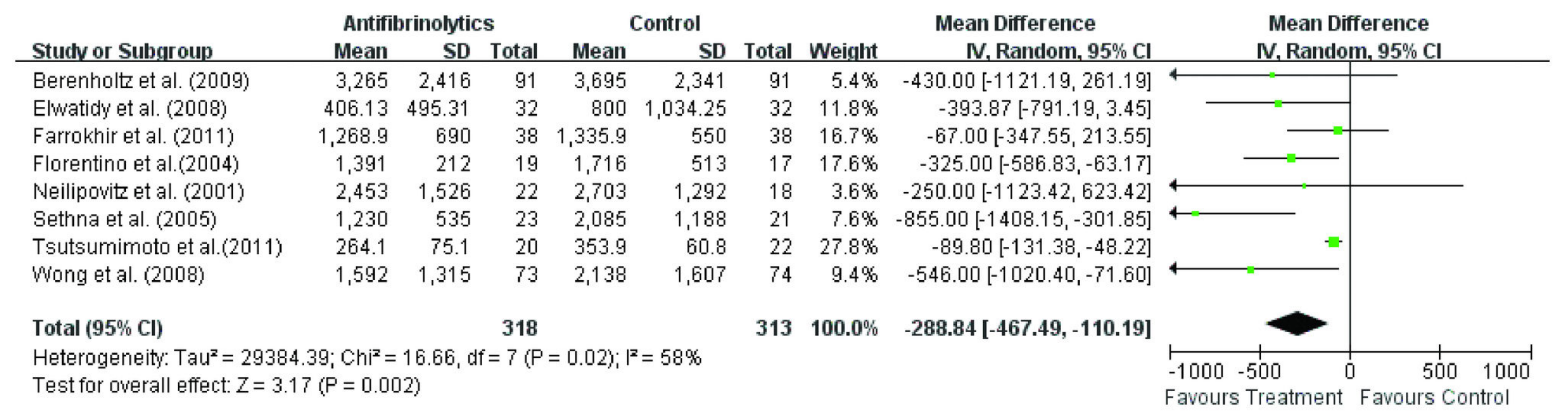
The weighted mean difference (WMD) estimate for total blood loss.

**Figure 4:**
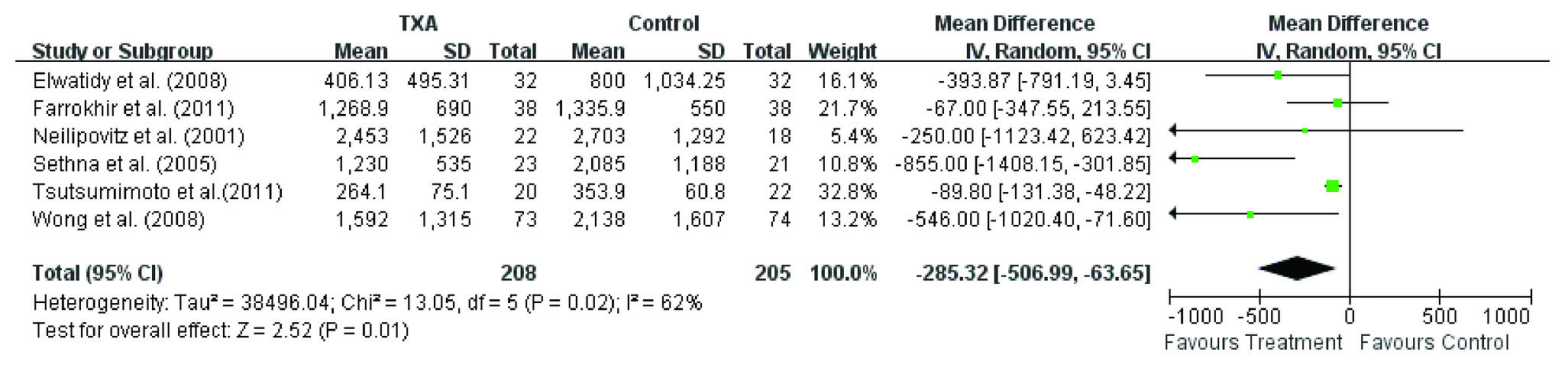
The weighted mean difference (WMD) estimate for total blood loss.

**Figure 5:**
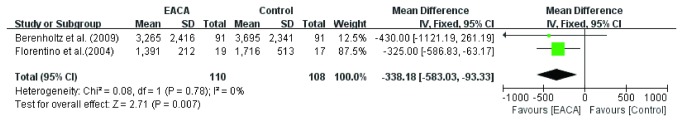
The weighted mean difference (WMD) estimate for total blood loss.

Total blood loss was available in eight studies [3,11-16,18], while one study didn’t provide the standard deviation [17]. Random effect model was used to analyze the pooled data. The overall effect showed difference (WMD =-288.8, 95 % CI – 46.49, - 110.19; *P* = 0.002) in total blood loss between the two groups (Figure 3). The subgroup analysis of six studies [3,11-15] involving TXA on total blood loss showed significant difference (WMD =-285.3, 95 % CI – 506.99, - 63.65; *P* = 0.01) in total blood loss between TXA group and control group (Figure 4). Also, the subgroup analysis of two studies [16,18] involving EACA on total blood loss showed less total blood loss (WMD =-338.1, 95 % CI – 583.03, - 93.33; *P* = 0.007) in EACA group (Figure 5), and the difference was more significant than TXA group. The heterogeneity in EBL may be caused by EBL methods, the differences of operation procedures and so on. 

#### Blood transfusion

**Figure 6:**
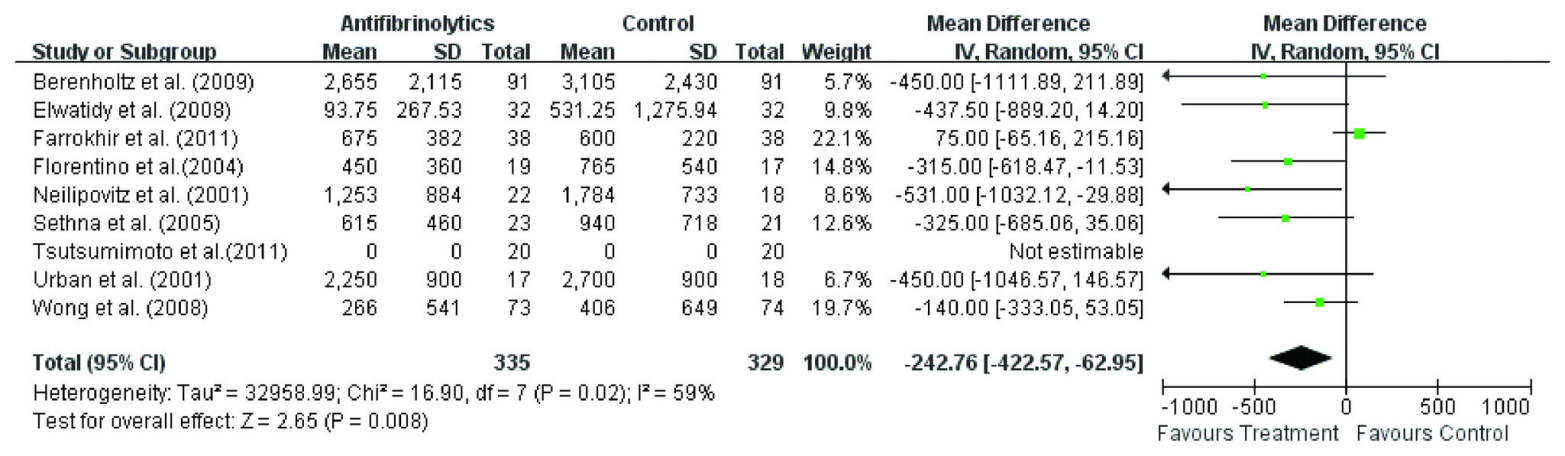
The weighted mean difference (WMD) estimate for blood transfusion.

**Figure 7:**
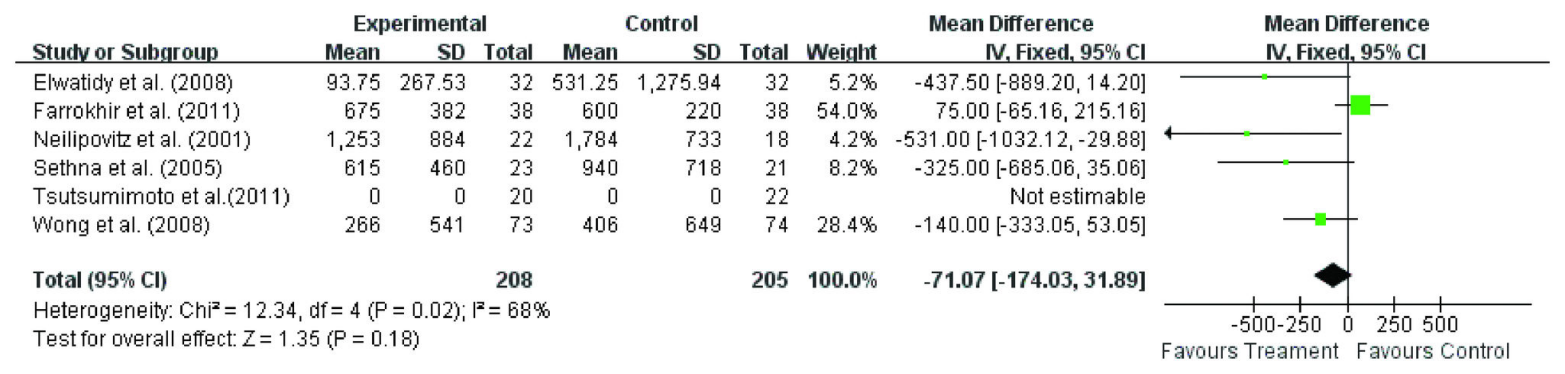
The weighted mean difference (WMD) estimate for blood transfusion.

**Figure 8:**
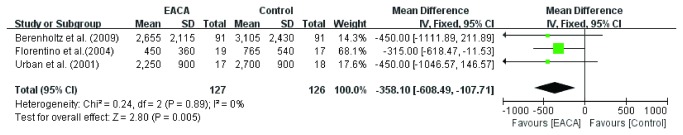
The weighted mean difference (WMD) estimate for blood transfusion.

Total blood transfusion was available in all the nine studies [3,11-18]. Random effect model was used to analyze the pooled data. The overall effect showed significant difference (WMD =-242.76, 95 % CI – 422.5, - 62.95; *P* = 0.008) in total blood transfusion between the two groups (Figure 6). The subgroup analysis of six studies [3,11-15] involving TXA showed significant difference (WMD =-242.76, 95 % CI – 422.5, - 62.95; *P* = 0.008) in total blood transfusion between TXA group and control group (Figure 7). Also, the subgroup analysis of three studies [16-18] involving EACA less total blood transfusion (WMD =-358.1, 95 % CI – 608.49, - 107.71; *P* = 0.005) in EACA group (Figure 8) and the difference was more significant than TXA group. As far as we are concerned, the cause of this heterogeneity is probably the difference of transfusion indication.

#### Ratio of blood transfusion

**Figure 9:**
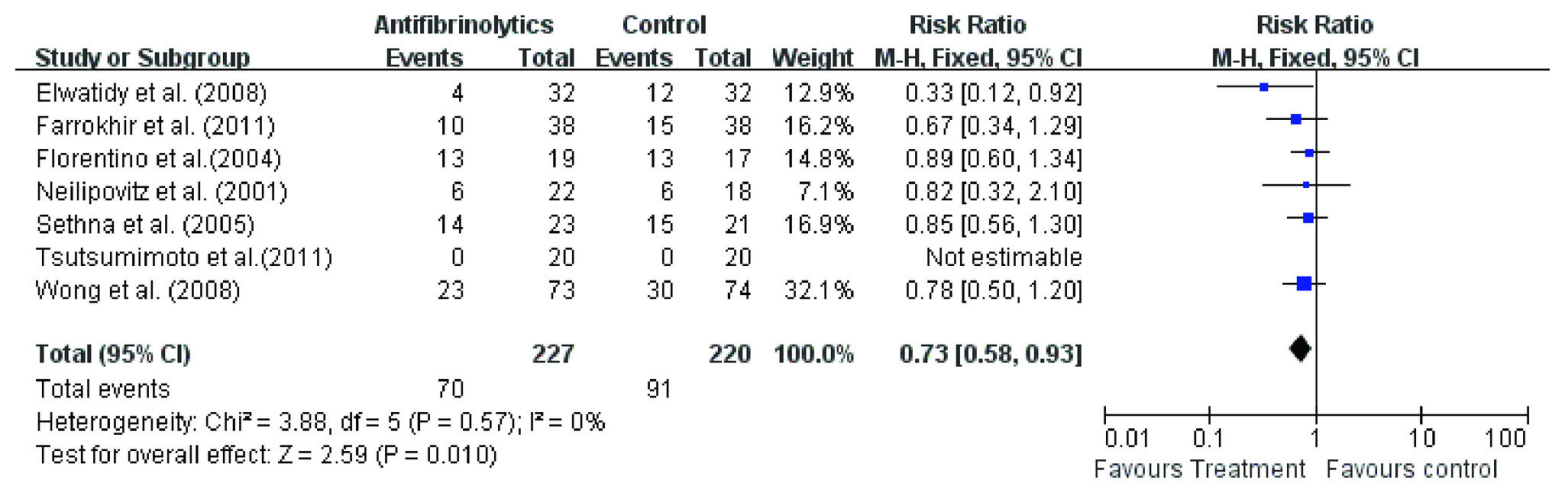
The risk ratio (RR) estimate for ratio of blood transfusion.

**Figure 10:**
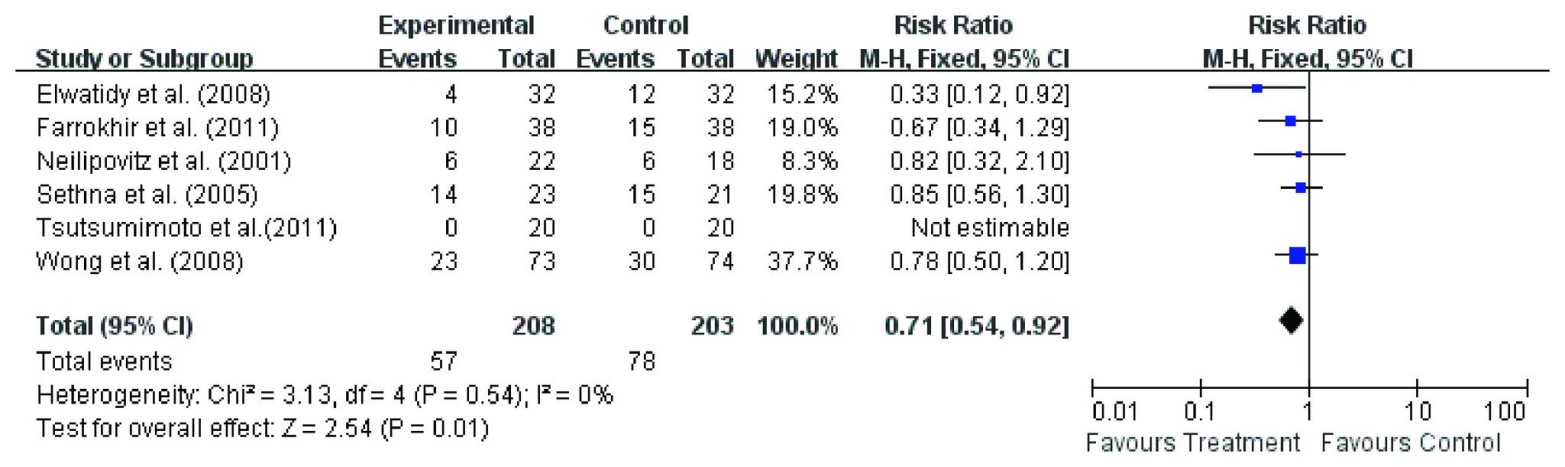
The risk ratio (RR) estimate for ratio of blood transfusion.

Ratios of blood transfusion were available in seven of the nine studies [3,11-16]. Fixed effect model was used to analyze the pooled data. The RR for ratio of blood transfusion of experiment groups was 0.73 (95% CI 0.58, 0.93; *p* = 0.010) compared with control groups (Figure 9). The result showed a ratio of blood transfusion in 70 of 227 patients in experiment groups and 91 of 220 patients in control groups. The subgroup analysis of six studies [3,11-15] involving TXA also showed that the ratio of blood transfusion in experiment group was significant lower(RR =0.71, 95 % CI 0.54, 0.92; *P* = 0.01) than control group (Figure 10).

#### Incidence of DVT

**Figure 11:**
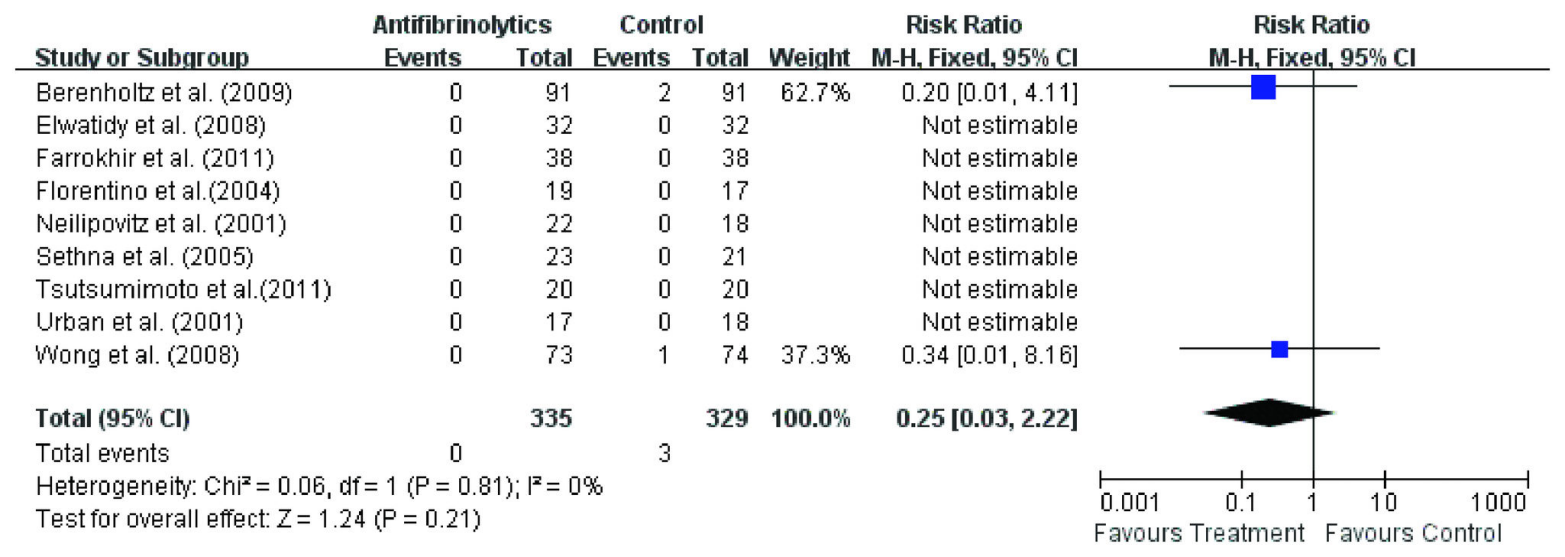
The risk ratio (RR) estimate for incidence of deep vein thrombosis.

Incidence of DVT were available in all the nine studies [3,11-18]. Fixed effect model was used to analyze the pooled data. There was no significant difference (RR 0.25, 95 % CI 0.03, 2.22; P = 0.21) in the incidence of DVT in the two groups (Figure 11). The result showed an incidence of DVT in 0 of 335 patients in experiment groups and 3 of 329 patients in control groups. 

## Discussion

All the nine studies were RCTs, and eight studies were double-blinded except the one carried by Tsutsumimoto et al [14]. All the eight studies did well in blinding of outcome assessment such as measuring EBL. Patients with coagulation disorders or using anticoagulant drugs were excluded in three studies [12,14,15]. The results of the present study showed that antifibrinolytic agents (as well as subgroup analysis of TXA, EACA) significantly reduced the total blood loss and blood transfusion in patients received spine surgery, and the result showed EACA was more efficacy than TXA. Also, the result confirmed that there were no increased incidences of DVT related to the use of antifibrinolytic agents. The results of our study were consistent with studies carried out by Zufferey et al [19].

The use of antifibrinolytics has provoked concerns about increased complications, especially the increased thrombotic tendency. The potential of postoperative thrombosis needs to be more carefully explored and diligent reporting of all adverse events must be adopted [5]. Dunn et al [20] performed a review of TXA in spine surgery, complications of cerebral thrombosis, arterial thrombosis, acute renal failure, and coronary graft occlusion was all reported. Case reports of thrombus formation on pulmonary artery catheters existed in patients receiving EACA [21]. The meta-analysis in 2007 performed by Henry et al [22] found the increase of myocardial infarction in aprotinin group. However, the meta-analysis updated in 2011 also performed by Henry et al demonstrated aprotinin resulted in a significant increase in the risk of death and a non-significant increase in the risk of myocardial infarction [23]. Urban et al [17] found aprotinin might elicit an anaphylactic reaction with repeated administration. In addition, their research was directed at identifying those cytokines responsible for pulmonary injury and whether aprotinin could block this response. Aprotinin was withdrown in 2007 because of safety concerns since Mangano’s [24] study in cardiac surgery. So the drug is not available anymore for daily clinical practice and two RCTs [25,26] about aprotinin were not included in our meta-analysis.

Antifibrinolytics are used to decrease perioperative blood loss and transfusion requirements through the inhibition of clot degradation. For more than 40 years, these medications have been used in cardiac and major orthopaedic surgery with proven efficacy [27]. Yagi et al [28] demonstrated intravenous TXA appeared to be safe and effective in posterior spinal fusion for adolescent idiopathic scoliosis. Similarly, Thompson et al [29] and Dhawale et al [30] demonstrated the efficacy of EACA in patients with scoliosis undergoing spine fusion. A meta-analysis in2008 performed by Gill BJ et al [31] indicated that antifibrinolytics were effective in reducing blood loss and transfusions in spine surgery, and epsilon-aminocaproic acid had a better effect compared with the other two agents, though the difference was not significant. However, the main limitations of that meta-analysis were the quality of the included studies (including NRCTs) and the lack of analysis on DVT incidence rate.

Using antifibrinolytic agents is safer compared to blood transfusion, though antifibrinolytic agents are expensive [32].The dose of atifibrinolytic agents used in spine surgery is controversial, though Karski et al [31] thought large dose could bring better efficacy. There were two main limitations of our meta-analysis. One was the confounding factors which disturbed the outcomes. The heterogeneity was remarkable of variation in drug dose, surgery procedure, operation time, age of patients, protocol for estimating blood loss and transfusion indication. There were no common criteria for estimating blood loss, so it was difficult to prevent the bias caused by estimating blood loss. And we couldn’t ensure the blinding methods were correct and true. Another limitation was that all the studies had very low subject numbers in each group(＜40per group) with the exception of Wong et al [12] and Berenholtz et al [18]. Furthermore, no authors provided further information, although we had tried to contact some of them. We extracted the data directly from the article. More high quality randomized prospective studies with larger sample size and complete data are required for further meta-analysis.

## Conclusion

In conclusion, it cannot be definitively concluded whether antifibrinolytic agents are safe, but it can be concluded antifibrinolytic agents are efficacy in spine surgery.

## Supporting Information

Checklist S1 **PRISMA 2009 Checklist for the Meta-Analysis.**
(PDF)

Flow Diagram S1 **The study selection and inclusion process.**
(DOC)
