# Supplementary material for: Efficacy and Safety of Using Antifibrinolytic Agents in Spine Surgery: a Meta-Analysis
Source: PLoS One. 2013 Nov 22;8(11):e82063. doi: 10.1371/journal.pone.0082063 (PMC3838357; doi:10.1371/journal.pone.0082063)
Supplement: Flow Diagram S1 — The study selection and inclusion process. (DOC) [file pone.0082063.s002.doc]

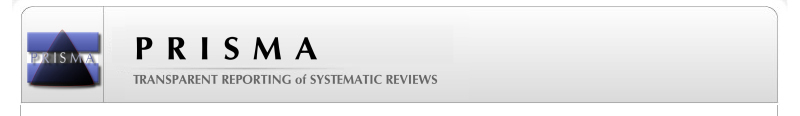
**PRISMA 2009 Flow Diagram**

**Screening**

**Included**

**Eligibility**

**Identification**

Records identified through database searching
(n =396 )

Additional records identified through other sources
(n =0 )

Records after duplicates removed
(n =256 )

Records screened
(n =256 )

Records excluded
(n = 215 )

Full-text articles assessed for eligibility
(n =41 )

Full-text articles excluded, with reasons
(n =32 )

Studies included in qualitative synthesis
(n =9 )

Studies included in quantitative synthesis (meta-analysis)
(n =9 )
